# Near-infrared multispectral photoacoustic analysis of lipids and intraplaque hemorrhage in human carotid artery atherosclerosis

**DOI:** 10.1016/j.pacs.2024.100636

**Published:** 2024-07-22

**Authors:** Jonas J.M. Riksen, Sowmiya Chandramoorthi, Antonius F.W. Van der Steen, Gijs Van Soest

**Affiliations:** a Department of Cardiology, Thorax Center, Cardiovascular Institute, Erasmus MC University Medical Center, Rotterdam, the Netherlands; b Verasonics Inc, Kirkland, WA, USA; c Department of Imaging Science and Technology, Delft University of Technology, Delft, the Netherlands; d Department of Precision and Microsystems Engineering, Delft University of Technology, Delft, the Netherlands; e Wellman Center for Photomedicine, Massachusetts General Hospital, Boston, MA, USA

**Keywords:** Photoacoustic imaging, Multispectral analysis, Carotid artery atherosclerosis, Atherosclerotic plaque

## Abstract

Spectral photoacoustic imaging in combination with unmixing techniques may be applied to retrieve information about high-risk features present in atherosclerotic plaques, possibly providing prognostic insights into future stroke events. We present the photoacoustic spectral contrast found in 12 systematically scanned advanced atherosclerotic plaques in the near-infrared wavelength range (850–1250 nm). The main absorbers are lipid, water, and hemoglobin, with the highest photoacoustic intensities at the lipid’s second overtone at 1190 and 1210 nm. Linear unmixing resulted in visualizing regions with high lipid and hemoglobin absorption, corresponding to the histological presence of lipid and intraplaque hemorrhage. A non-negative matrix factorization approach reveals differences in lipid spectral contrast, providing potential insights into the vulnerability of atherosclerotic plaque. These results provide a reference for future, more complex, *in vivo* photoacoustic imaging of carotid artery atherosclerosis, potentially contributing to assessing the risk of future events and treatment decision.

## 1 Introduction

Stroke is a leading cause of death and disability. The American Heart Association estimates that around 795,000 people in the United States suffer a stroke every year [1]. Often, stroke is caused by carotid artery atherosclerosis, in which inflammation and lipid accumulation leads to plaque formation. Destabilization of the plaque releases thrombogenic content into the bloodstream, which leads to vessel occlusion in the brain [2]. Strokes caused by atherosclerosis can be prevented if unstable carotid atherosclerotic plaques can be detected early and managed appropriately [3], [4]. An important step towards achieving this involves imaging so-called high-risk atherosclerotic plaque features, which are predictive of unstable plaques and ultimately stroke. Two high-risk plaque features are the presence of intraplaque hemorrhage and specific plaque lipids [5], [6]. Therefore, enhancing imaging may improve risk assessment and inform treatment decisions. However, the current clinically used imaging modalities, such as ultrasound (US) and x-ray computed tomography (CT), lack chemical specificity and the assessment of high-risk plaque is therefore limited to morphological information.

Photoacoustic imaging (PAI) is an ultrasonic imaging modality with sensitivity for optical absorption [7]. Optical contrast obtained by differential absorption of various light wavelengths by specific chromophores provides functional or molecular imaging. Imaging targets include hemoglobin (Hb), melanin, various lipids, or contrast agents. PAI has been explored for clinical use over the past decade [7], [8], [9] in, among others, visualizing vascular structures and oxygenation [10], inflammation [11], [12], and lymphatic vessel imaging [13], [14]. It is frequently used in combination with US imaging.

Atherosclerotic plaques are heterogeneous structures consisting of a multitude of fibrous, lipidic, calcific, and cellular structures. A multispectral photoacoustic imaging (sPAI) approach with a range of wavelengths may be applied to retrieve the various absorption spectra pertaining to different molecular constituents of plaque. This operation is referred to as spectral unmixing, and a variety of algorithms have been proposed to analyze spectra of the different absorbers present in each image pixel [15]. Supervised spectral unmixing, using linear unmixing models, estimates relative concentrations using linear regression [16]. Unsupervised spectral unmixing methods, or “blind” unmixing, do not rely on information based on prior knowledge of the targeted spectra or background and aim to separate distinct spectra within the mixed dataset by modeling the data and solving an optimization problem [17], [18].

Previous work has shown *in vivo* PAI of the carotid artery in healthy volunteers, showing the possibility of noninvasive handheld sPAI imaging [19], [20]. This has been demonstrated in several studies to characterize intraplaque hemorrhage and lipid composition in human carotid plaque. Imaging in the wavelength range of 800–950 nm captured intraplaque hemorrhage in several *ex vivo* [21], [22] and intraoperative studies [23], where intraplaque hemorrhage showed different characteristics from luminal blood. The lipid absorption peak at approximately 930 nm has been used to identify lipids in plaque *in vivo* [24]. Several *ex vivo* studies also demonstrated the use of the more strongly absorbing 1200 nm absorption band to image lipids in human atherosclerotic arteries [25], [26], [27]. In this wavelength range, the strong attenuation of the skin and subcutaneous fat hinders an external illumination approach [28]. To account for this, an alternative interstitial illumination approach has been demonstrated to facilitate the use of this absorption band for in-vivo carotid artery atherosclerosis PAI [29]. Besides the greater optical absorption, this spectral band has also shown its ability to differentiate between different lipid types, based on distinct lipid absorption spectra [30]. In excised human carotid artery endarterectomy (CEA) specimens, microspectroscopic PAI also showed that plaque lipid photoacoustic signals exhibit distinct spectral features, which can be used to identify different lipid plaque signatures. These features result from differences in molecular structure, primarily hydrocarbon chain length and the degree of saturation [31]. High photoacoustic intensity areas have been found to co-locate with the presence of cholesteryl ester (18:2) and sphingomyelin (34:1), which are abundant in more advanced atherosclerotic plaques and particularly in areas of necrosis [5], [31]. Therefore, this spectral lipid contrast may be a source of predictive information on plaque vulnerability. Nevertheless, it has not yet been established whether these lipid signatures can be measured in a tomographic fashion in intact atherosclerotic plaque.

In this study, we provide a multispectral photoacoustic analysis of carotid artery atherosclerosis in the wavelength range from 850 to 1250 nm. We highlight the photoacoustic spectral contrast found in 12 systematically scanned advanced atherosclerotic plaques, providing information useful for optimal wavelength selection for *in vivo* scanning. The first objective is to show the detection of intraplaque hemorrhage and high lipid areas and how these relate to histology. The second objective is to go beyond the simple presence of lipid and explore whether the spectral lipid contrast previously found in microspectroscopic PAI can also be discriminated in intact carotid plaques. Compared to previous studies of human carotid atherosclerosis [21], [22], [23], [24], [25], [26], [27], [29], this work adds a comprehensive, broadband spectroscopic characterization of plaque features, distinguishing intraplaque hemorrhage and spectrally distinct lipid areas, revealing novel discriminative features. These spectral features could be used for future *in vivo* detection of advanced atherosclerotic plaque features that may improve the prediction of plaque instability and stroke.

## 2 Methods

The sPAI was performed on 12 human CEAs, obtained from patients with symptomatic stenosis (≥ 50 %), using the inVision 256-TF (iThera Medical GmbH, Munich, Germany). A stenosis is classified as symptomatic if it has resulted in ischemic events in the eye or cerebral hemisphere within the preceding six months [32]. After scanning, the sample was embedded, sectioned, and stained for histological comparison. The tissue collection was performed according to a protocol approved by the Medical Ethics Board at the Erasmus MC, University Medical Center Rotterdam (MEC 2008-147). Prior to inclusion, all subjects provided written informed consent.

### 2.1 Tissue handling

Directly after the CEAs were surgically excised, they were snap-frozen and preserved at − 80 °C. Before scanning, the CEAs were thawed to room temperature in phosphate-buffered saline and suture wires were added to the outer ends for later registration between the photoacoustic images and histology. After sPAI scans, the CEAs were cut into 3 mm blocks, embedded into 10 % porcine type A gelatin (Sigma-Aldrich, The Netherlands), and stored for later cryosectioning. The 10 µm cryosections were histologically stained with Oil Red O (ORO) (Sigma-Aldrich, The Netherlands), and Martius Scarlet Blue (MSB) (Martius Yellow, Brilliant Crystal Scarlet, Aniline Blue) (Sigma-Aldrich, The Netherlands), at three different locations within each CEA.

### 2.2 Spectral photoacoustic imaging

The CEA samples were imaged in the imaging tank within the designated holder and 7 µm plastic foil, serving as a coupling membrane between the sample and tank. To facilitate acoustic coupling, the plastic foil surrounding the sample and the imaging chamber were filled with deuterium oxide (heavy water; D_2_O). The absorption in the near-infrared (NIR) wavelength range (see Supplementary Fig. 1) would cause a differential attenuation in the excitation light of different wavelengths when regular water is used. This, in turn, influences the relative response of the CEA sample across different wavelengths, complicating spectral unmixing. The absorption spectrum of D_2_O is blueshifted relative to that of water and thus is relatively lower and featureless in the NIR. It avoids so-called spectral coloring caused by water or ultrasound gel [33].

The samples were positioned in the center of the imaging chamber and tomographic US array, with 360° illumination (10 Hz, pulse energy up to 70 mJ), and 270° acoustic detection (5.0 MHz center frequency, > 55 % bandwidth). sPAI was performed at wavelengths from 850 to 1250 nm, with a spectral resolution of 5 nm, and averaged five times. Cross-sections were scanned in steps of 1 mm from the proximal to the distal suture wire, resulting in imaging times of ∼ 10 min.

Image reconstruction was carried out using the viewMSOT software (iThera Medical GmbH, Munich, Germany) using a back-projection algorithm [34] with a field of view of 20 mm (72 µm cross-sectional resolution), with low and high filter cut-off frequencies of 50 kHz to 6.5 MHz.

### 2.3 Spectral unmixing

Two unmixing techniques were applied, each with its own objective. Firstly, to identify regions of lipid and intraplaque hemorrhage within the sample, the viewMSOT linear regression algorithm was employed, where spectral pixel intensities are fitted with a linear combination of reference spectra [35]. The reconstructed spectral images (850–1250 nm) were unmixed using the normalized reference spectra for water, lipid, and HbO_2_ (Supplementary Fig. 1) from the software.

Secondly, unsupervised non-negative matrix factorization (NNMF) was applied to identify the presence of different lipid spectra in the lipid absorption band around 1200 nm [36]. For this, the reconstructed spectral photoacoustic images were exported to MATLAB (R2020b, The MathWorks Inc., MA, USA), where the matrix for factorization was composed by including wavelengths between 1150 and 1250 nm, to emphasize the spectral variation in this range, and pixels from the twelve CEA samples. For the pixel selection, the CEAs were manually segmented per cross-section to reduce the inclusion of reconstruction artifacts and the (minimal) absorption from the plastic foil. Subsequently, thresholding was applied (intensity > 200 a.u.) to limit the effect of noisy pixels. A total of ∼ 2.380.000 pixels were included. The final preprocessing step involved normalizing through the standardization of the area under the curve (AUC = 1) of spectral pixels. Compared to min-max normalization, this method is less sensitive to extreme values and outliers, preserving the relative relationships and proportions within the spectrum when compared to a min-max normalization approach. Because of the unknown number of distinct spectra, the number of end members was incrementally raised until just before the onset of noise unmixing. The resulting unmixed spectrum per NNMF component is plotted and the weights for each pixel are visualized in abundance maps to show the spatial distribution of each NNMF component.

## 3 Results

Lipid, water, and Hb are the dominant chromophores (see reference spectra in Supplementary Fig. 1); these can be found in all CEA specimens in varying compositions. Other chromophores (like collagen) were not detectable in this wavelength range. Supplementary Fig. 2 collects the mean spectra from all samples. The highest photoacoustic signals are observed at lipid absorption peaks at 1190 nm and 1210 nm.

### 3.1 Detection of lipid and intraplaque hemorrhage using linear unmixing

**Fig. 1:**
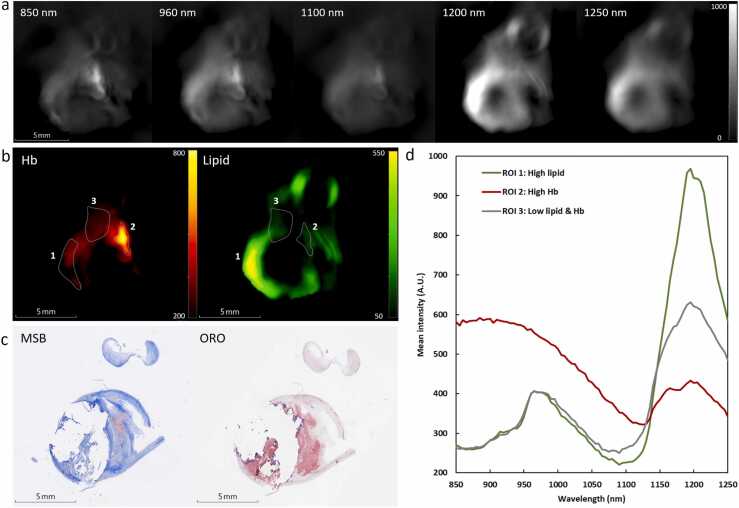
sPAI images and histology of a cross-section with a lipid core and intraplaque hemorrhage. The cross-section at different wavelengths is shown in (a), with the unmixed hemoglobin (Hb) and lipid image in (b) and three regions of interest (ROI): High lipid, high Hb, low lipid & Hb. (c) shows the corresponding histology stained with MSB (left) staining fibrin red and collagen blue, and ORO (right) staining lipids red, showing intraplaque hemorrhage, a lipid core, and lipid throughout the sample. (d) shows the mean photoacoustic spectra of the ROI in (b).

We extracted information about plaque composition in terms of the lipid, water, and Hb constituents. Fig. 1 shows a cross-section of a typical CEA sample, where it shows that wavelengths around 1200 nm generate most photoacoustic signal (Fig. 1a). The unmixed Hb and lipid images in Fig. 1b highlight locations with a high concentration of Hb and lipid and match the histology findings in Fig. 1c, where intraplaque hemorrhage is stained by MSB and lipid by ORO.

**Fig. 2:**
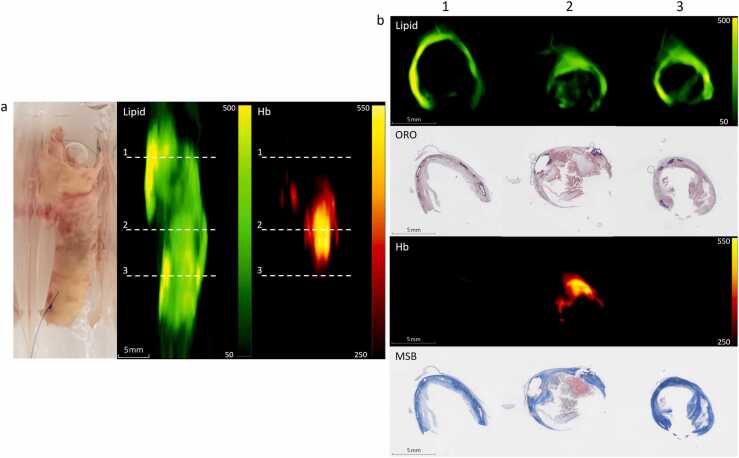
(a) Top view of a scanned CEA sample with left a photo, middle maximum intensity projection (MIP) of unmixed lipid, and right MIP of hemoglobin (Hb). The dashed lines indicate the locations of the three cross sections throughout the sample visualized in (b), from top to bottom: unmixed lipid, ORO staining lipids in red, unmixed Hb, and MSB staining fibrin in red.

Fig. 2a shows a typical lipid and Hb distribution throughout a CEA sample, where the spectrally unmixed lipid values are dispersed over the sample with certain regions exhibiting higher values. Fig. 2b shows cross-sections throughout the sample along with the corresponding histology. In the corresponding ORO-stained histology slices, it can be noted that the red-stained lipid areas correspond to the locations with higher lipid values. The spectrally unmixed Hb values are primarily concentrated in the second cross-section. This corresponds to the location of the MSB-stained fibrin, indicating the location of intraplaque hemorrhage.

**Fig. 3:**
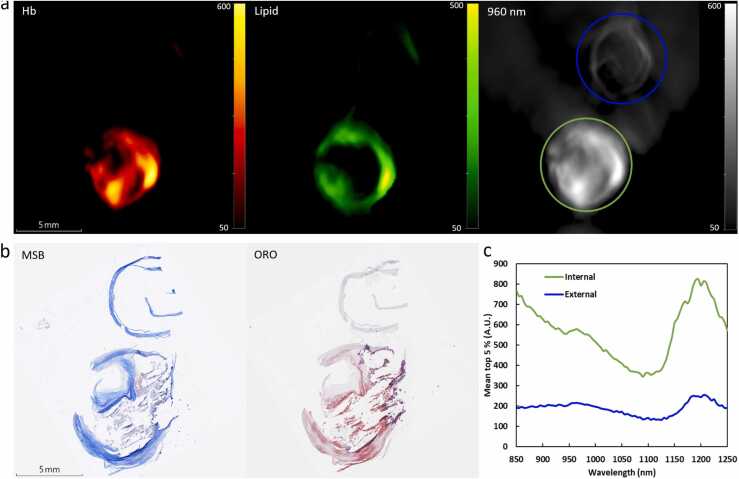
(a) Cross-section of unmixed Hemoglobin (Hb), unmixed lipid, and 960 nm with regions of interest (ROI) for the normal external (blue) and the atherosclerotic internal part (green) of the CEA. (b) Corresponding histology showing the atherosclerotic internal carotid artery with MSB staining fibrin in the intraplaque hemorrhage in red (left) and ORO staining lipids red (right), amongst the visible calcification and cholesterol deposits. (c) Mean photoacoustic spectra (5 % highest pixels) from the ROI from the external and internal parts of the CEA from (a).

A bifurcated CEA sample cross-section is shown in Fig. 3. The internal carotid artery exhibits advanced disease, showing intraplaque hemorrhage, a lipid core with cholesterol deposits, and calcifications (Fig. 3b), while the external carotid artery has a thin wall and no signs of atherosclerotic changes. The different typical representation in unmixed signal is shown in Fig. 3a, with Fig. 3c showing the typical difference in photoacoustic spectra.

### 3.2 Unsupervised spectral unmixing of variation in lipid spectra

**Fig. 4:**
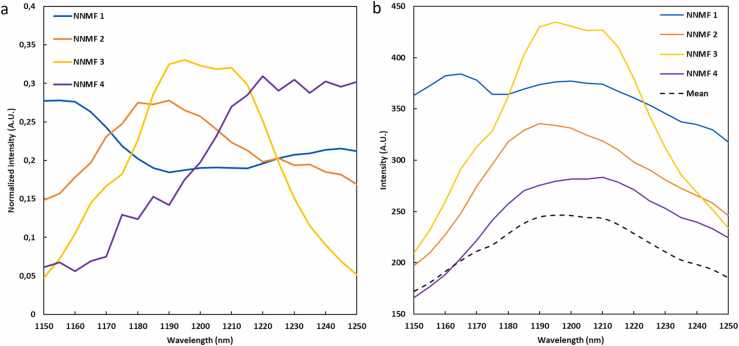
(a) Unmixed spectra by means of NNMF in the wavelength range of 1150–1250 nm from all 12 CEA samples. (b) Mean photoacoustic spectra from the pixels corresponding to the highest 10 % weights per NNMF component along with the mean spectrum from all pixels (dashed line).

The spectra unmixed by NNMF in the wavelength range of 1150–1250 nm highlight salient spectral features in the lipid absorption band. The spectra resulting from the analysis of all 12 CEA samples are presented in Fig. 4a. Fig. 4b shows the corresponding average photoacoustic spectra, without the normalization, from the pixels with the 10 % highest NNMF weights per NNMF component. It shows the presence of lipid spectra with peak intensities at wavelengths 1190 nm (NNMF 2), 1210 nm (NNMF 4), or both (NNMF 3). On average, the lipid pixels characterized by a dominant peak at 1190 nm exhibit a higher intensity compared to those with a dominant 1210 nm peak.

**Fig. 5:**
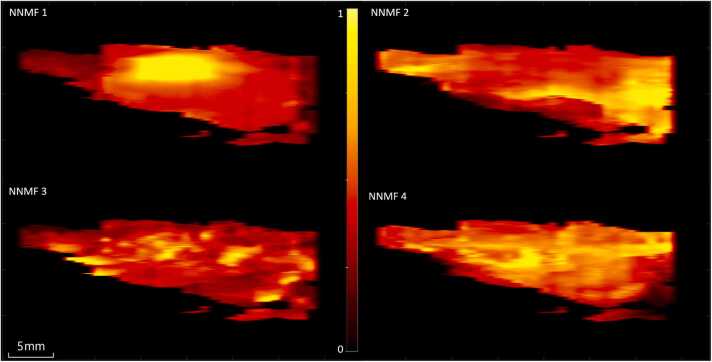
Maximum intensity projection of the abundance map from each NNMF component from a top view of a CEA sample.

**Fig. 6:**
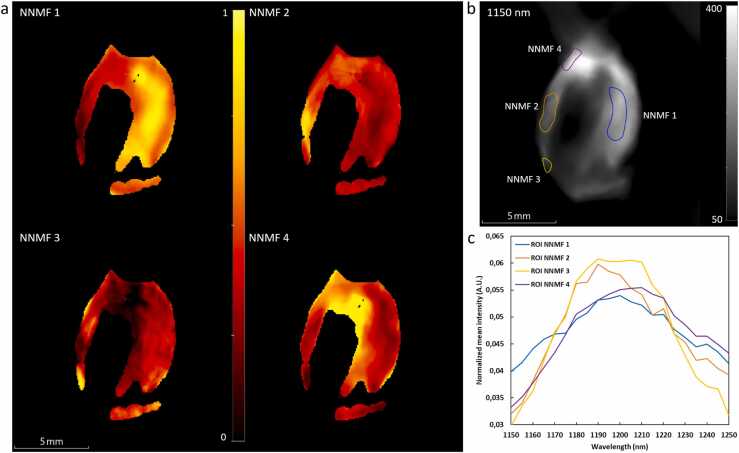
Cross-section with areas of high NNMF weights for all four NNMF components. (a) Abundance maps of the different NNMF components. (b) Photoacoustic image at 1150 nm with drawn regions of interest (ROI) at locations with high weights for their respective NNMF component. (c) Normalized mean photoacoustic spectrum per ROI from (b) (AUC = 1).

Fig. 5 shows maximum intensity projections (MIP) per NNMF component of a CEA sample. The different NNMF components tend to cluster throughout the imaged cross-sections and NNMF 1 mainly occurs in the center of more voluminous plaque regions. Fig. 6a shows a cross-section at a location with abundance maps from the four NNMF components. In this cross-section all NNMF components exhibit a region with a relatively high weight, allowing the comparison of spectral variability according to the dominant NNMF component. In Fig. 6b the photoacoustic image at 1150 nm is visualized with ROIs drawn to isolate these locations with relatively high values for each of the NNMF components seen in Fig. 6a. Fig. 6c presents the corresponding mean spectrum per ROI. This demonstrates that the unmixed spectra cluster and exhibit spectra resembling the NNMF spectra displayed in Fig. 4b.

## 4 Discussion

Our study presents a photoacoustic spectral analysis of 12 advanced CEA samples in the NIR wavelength range, with the capability of detecting and localizing lipids and intraplaque hemorrhage. Lipid, water, and Hb are the main absorbers with the second overtone region of lipids, around 1200 nm, exhibiting the highest photoacoustic signal peaks at 1190 nm and 1210 nm. In addition, we show the ability to measure various lipid spectra in intact atherosclerotic samples.

The first objective involved the detection of intraplaque hemorrhage and regions with a high lipid concentration and how these relate to histology. As mentioned in the introduction, the capability of PAI to observe carotid intraplaque hemorrhage [21], [22], [23] and lipids at 1200 nm [29] and 930 nm [24] has been demonstrated previously. Here, however, we systematically measure complete plaque samples in a broad NIR wavelength range (850–1250 nm), immersed in heavy water ensuring that photoacoustic signals only originate from the specimen. The linear unmixing approach, using reference spectra, resulted in visualizing regions with high lipid and Hb absorption. The high lipid areas corresponded to the ORO-stained lipid regions and the high Hb areas to the MSB-stained fibrin areas, indicating the locations of intraplaque hemorrhage (Fig. 1, Fig. 2, Fig. 3). Although our sample set only included advanced plaques with features of vulnerability, Fig. 3 shows the spectral difference between the internal carotid artery that is affected by atherosclerosis, compared to the normal external carotid artery segment, showing reduced presence of lipids and Hb in its respective spectra.

The second objective was to explore whether spectral lipid contrast can be discriminated in intact carotid plaques. Unsupervised spectral unmixing in the lipid absorption band ranging from 1150 to 1250 nm, resulted in four different NNMF components (Fig. 4). We selected the NNMF algorithm over other unsupervised methods because of its robustness and interpretable data [17]. Unmixing with more components resulted in the presence of noise in the analyzed spectra. The NNMF components 2, 3, and 4 unmix areas with a spectral lipid peak around 1190 nm (NNMF 2), lipid peaks at both 1190 and 1210 nm (NNMF 3), and a spectral lipid peak around 1210 nm (NNMF4). These observed differences in lipid spectra are caused by difference in molecular structure associated with hydrocarbon chain length and degree of saturation. The number of unsaturated bonds in lipids affects the physical chemistry of lipid deposits in biological systems and has been hypothesized to affect vulnerability [31], [37]. We also observe that lipid spectra with a dominant 1190 nm peak are in areas with higher photoacoustic intensity compared to spectra with a 1210 nm peak (Fig. 4, NNMF 2 and 4), as previously observed in a microspectral photoacoustic setup on CEA sections [31]. NNMF component 1 unmixes mainly spectrally-colored regions and is therefore primarily located in the center of the samples, where excitation light has traversed the more superficial tissue layers.

*In vivo* imaging of carotid atherosclerosis would benefit from the identification of lipid deposits and intraplaque hemorrhage. We observe in our linear unmixing studies that the hemoglobin signal near 900 nm can be reliably used to identify intraplaque hemorrhage. The wavelength range near 1100 nm consistently exhibits a spectral minimum. Lipids were detected at two distinct wavelengths, 1190 and 1210 nm. These wavelengths would be suitable for *in vivo* scanning, provided interstitial illumination [29] can be applied. Surprisingly, we were unable to detect a consistent signal associated with lipids, at 930 nm. This is at variance with previous findings in human atherosclerosis, that did report a lipid-specific signal in that wavelength band [24]. In contrast with this earlier work, we performed longitudinal scans with precisely matched histology analyses, providing a direct comparison between sPAI and tissue composition in which no spectral feature at 930 nm could be detected. However, subcutaneous adipose tissue shows a strong signal at 930 nm, and modulation of the signal at that wavelength has been associated with liver fat content [38], [39]. Our specimens all had a high water content and we hypothesize that spectral coloring may obscure the lipid signal, but further experiments are needed to confirm this.

The used imaging device exhibits the well-known limitation of 2D PA scanners, where strongly absorbing out-of-plane objects can produce artifacts. This may affect the spectroscopic image data and can result in an overestimation of the lipid and Hb volumes. The relatively low center frequency of the transducer leads to a lack of sensitivity for small-scale spatial variation in the atherosclerotic plaque, which in turn propagates into the spectral analysis.

It is worth noting that the sample set comprises plaques harvested from carotid endarterectomy. Calcification is a common feature of advanced plaque. The acoustic heterogeneity caused reconstruction artifacts. Additionally, calcifications complicate the histological sectioning, often leading to the removal of the calcified regions and deformations in the remaining histology slices. These limitations precluded a quantitative comparison between lipid or intraplaque hemorrhage area.

Image intensity and unmixed component (lipid/Hb) weight in the sPAI scans are assumed to reflect component abundance. In reality, they strongly depend on spectral coloring, and thus on sample thickness and the state of hydration of the sample. In addition, all CEA samples displayed positive ORO lipid staining. The absence of healthy arteries means we could not compare to independent controls.

For the unmixed Hb comparison, we find the MSB-stained fibrin collocates with areas with high Hb values. However, we also observed some areas with high Hb where no fibrin is stained in the corresponding histology. A possible explanation is the presence of remaining fresh red blood cells during imaging, which are flushed out of the sample before histological staining.

Spectral coloring may complicate the interpretation of NNMF spectra because of the significant absorption in the lipid wavelength range. This affects the pixels included in the NNMF analysis, and therefore the unmixed spectra and the contrast we observe could be attributed to spectral coloring. Nevertheless, we observe the same difference, evident in Fig. 6c NNMF2 and 3, in lipid spectral shapes in cross-sections with a thin vessel wall, samples, where the spectral coloring should be minimal (Supplementary Fig. 3), inspiring confidence in this result.

Preoperative *in vivo* imaging of carotid artery atherosclerosis using PAI has been previously demonstrated in the wavelength range of 700–970 nm [24]. However, primarily due to light attenuation, the depth of penetration was limited. Limited penetration depth is anticipated, yet this may be mitigated by an interstitial illumination approach that could enable *in vivo* spectroscopic lipid imaging [29]. Further technological advances in optical illumination and enhanced ultrasonic reception could improve the imaging depth. Nevertheless, spectral coloring complicates accurate spectroscopic assessment, which could be mitigated by fluence compensation [40], [41]. Additionally, when translating to an *in vivo* setting, motion of the carotid artery requires gated acquisition or motion correction. This work demonstrates the signals that can be expected in the absence of these translational challenges and could serve as a reference point for future *in vivo* carotid artery PAI.

## 5 Conclusion

In this study, we presented a multispectral photoacoustic analysis of 12 human CEAs in the NIR wavelength range (850–1250 nm) and the capability of detecting and localizing lipid and intraplaque hemorrhage. In addition, we demonstrated the presence of distinct lipid spectral features, measured in intact samples that possibly contain information about plaque vulnerability. We have shown the multispectral photoacoustic signals that emerge purely from carotid artery atherosclerosis that could act as a reference point for more complex *in vivo* PAI, which may offer insights about the plaque vulnerability and potentially aid in treatment decision and assessing the risk of future stroke.

## CRediT authorship contribution statement

**Jonas Riksen:** Writing – review & editing, Writing – original draft, Methodology, Investigation, Formal analysis, Conceptualization. **Gijs van Soest:** Writing – review & editing, Supervision, Methodology, Conceptualization. **Antonius Van der Steen:** Writing – review & editing, Supervision. **Sowmiya Chandramoorthi:** Writing – review & editing, Methodology, Investigation, Conceptualization.

## Declaration of Competing Interest

The authors declare the following financial interests/personal relationships which may be considered as potential competing interests: Gijs van Soest is a cofounder of, and has equity in, Kaminari Medical BV, who were not involved in the submitted work. In the past three years, he was the PI on research projects, administered by Erasmus MC, that received research support from FUJIFILM VisualSonics, Shenzhen Vivolight, Boston Scientific (outside the submitted work), Waters and Mindray (related to the submitted work). Ton van der Steen is a strategic advisor of, and has a financial interest in, Kaminari Medical BV. The other authors have no conflicts of interest to declare.

## Data Availability

The authors do not have permission to share data.
