# Supplementary material for: Near-infrared multispectral photoacoustic analysis of lipids and intraplaque hemorrhage in human carotid artery atherosclerosis
Source: Photoacoustics. 2024 Jul 22;38:100636. doi: 10.1016/j.pacs.2024.100636 (PMC11320465; doi:10.1016/j.pacs.2024.100636)
Supplement: Supplementary file 1 — Supplementary material. [file mmc1.docx]

# Supplementary figures


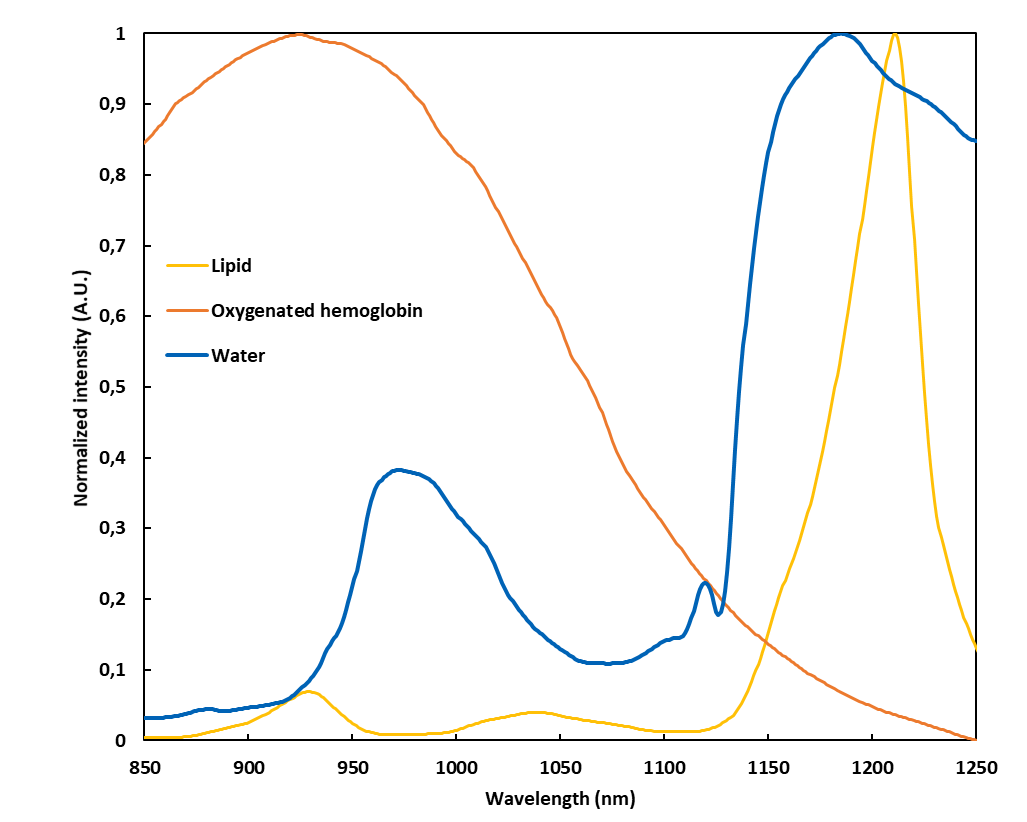


Supplementary Figure 1: Normalized reference spectra for lipid, oxygenated hemoglobin, and water that are used for spectral unmixing.


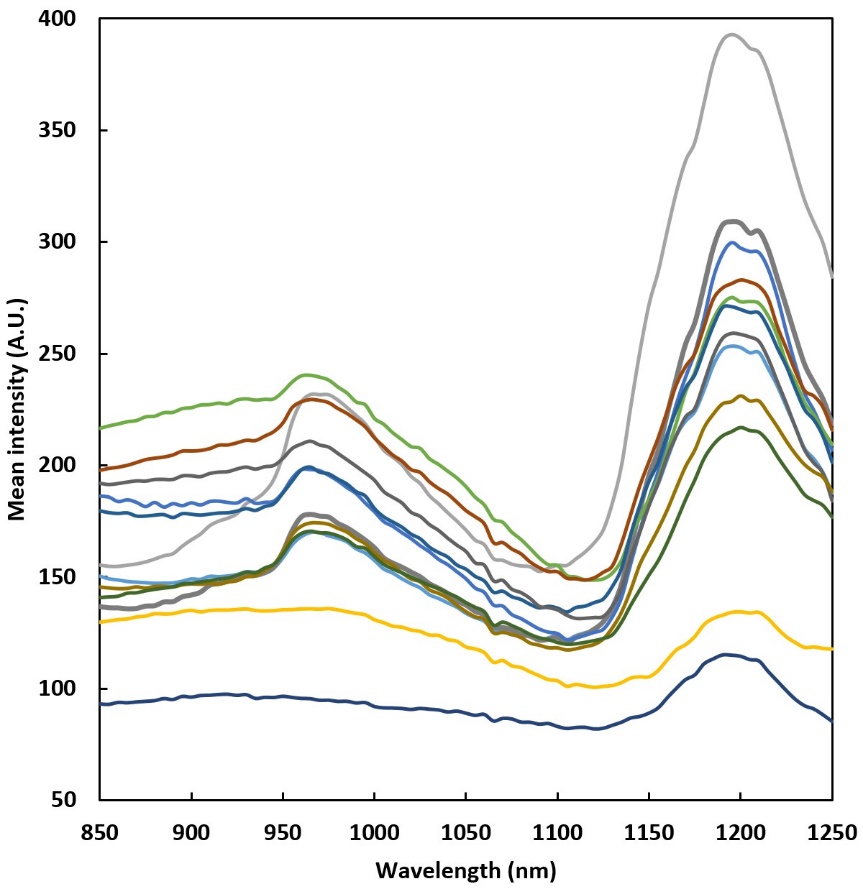


Supplementary Figure 2: Mean photoacoustic spectra of each individual (n = 12) carotid endarterectomy sample. Lipid, water, and hemoglobin are the dominant chromophores, present in varying relative concentrations. The highest photoacoustic signals are observed at lipid absorption peaks at 1190 nm and 1210 nm.


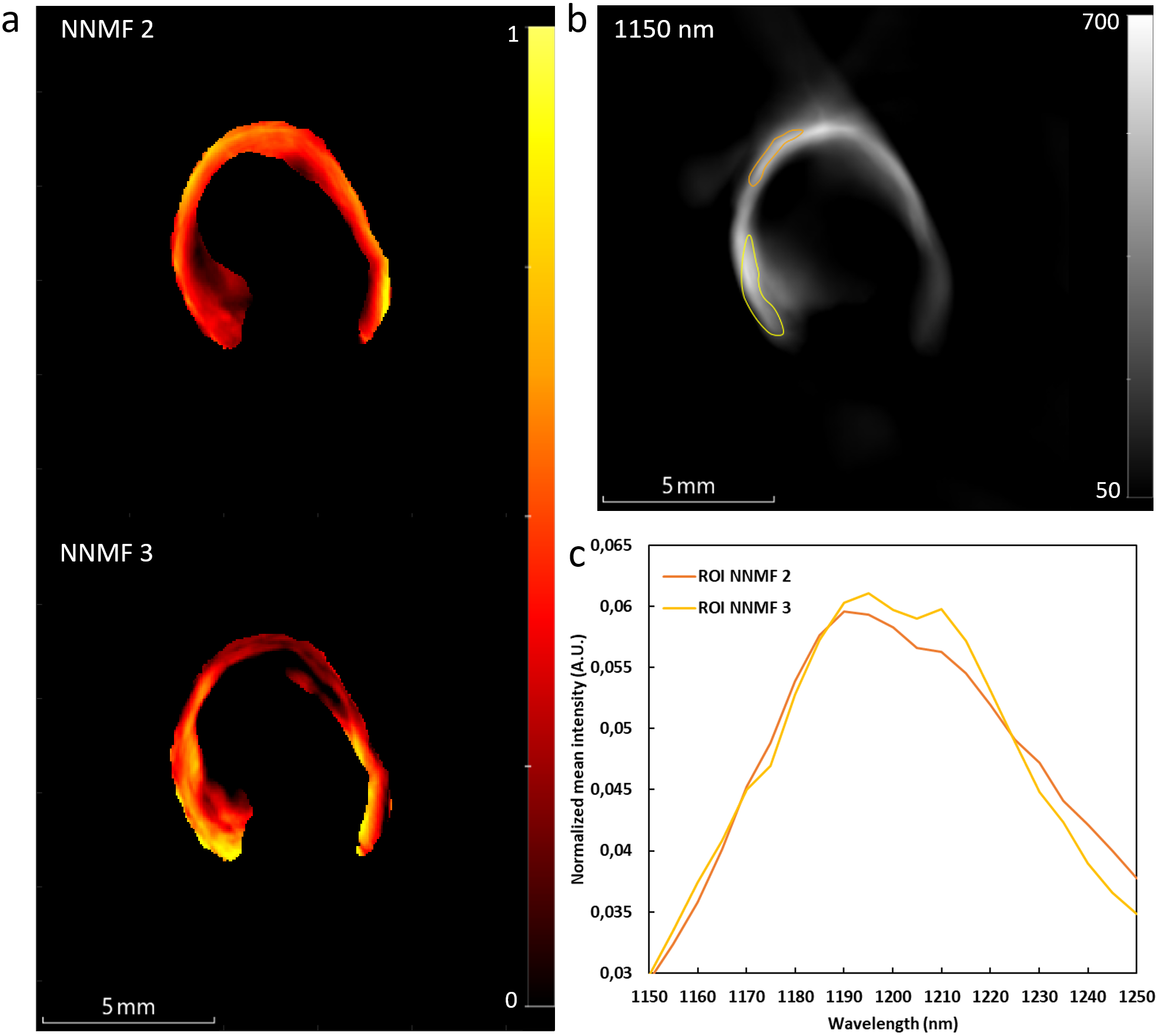


Supplementary Figure 3: Cross-section of a carotid endarterectomy where the sample is thin and therefore can be assumed to be minimally affected by spectral coloring. (a) Abundance maps of NNMF components 2 and 3. (b) Photoacoustic image at 1150 nm with drawn regions of interest (ROI) for each NNMF component. (c) Normalized mean photoacoustic spectrum per ROI from (b) (AUC = 1).
